# Supplementary material for: Identification of molecular nociceptors in Octopus vulgaris through functional characterisation in Caenorhabditis elegans
Source: Biol Open. 2026 Feb 2;15(1):bio062268. doi: 10.1242/bio.062268 (PMC12919962; doi:10.1242/bio.062268)
Supplement: Supplementary information [file biolopen-15-062268-s1.pdf]

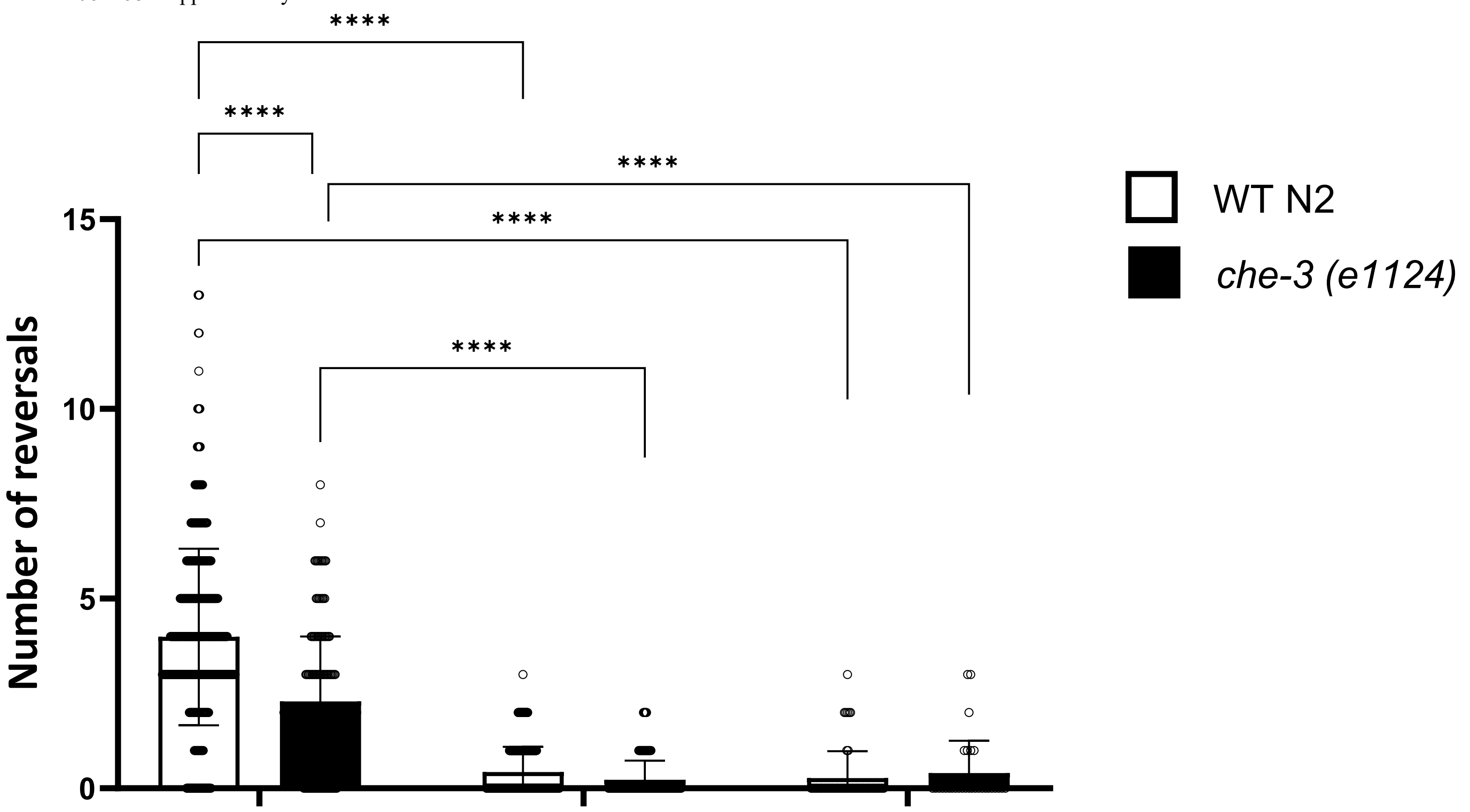

Fig. S1. *C. elegans* elicits a clear avoidance response when in contact with acidic pH in the drop assay. The bars represent the average number of reversals exhibited by worms ± SD. The assay highlights a clear aversive response to M9, pH3 in the WT N2 (3.990 ± 0.115 reversals) when compared to Ctrl M9, pH7 (0.432 ± 0.033 reversals, p<0.0001).

An effect of the repellents is still visible in che-3 (e1124) mutants in which the ultrastructural organisation of the sensory amphids is deficient (M9 pH3 vs Ctrl M9, pH7, p<0.0001) but a significant impairment in the response is evident when compared to the WT N2 (p<0.0001) with an average of 2.293 ± 0.130 reversals.

Additionally, worms were shown to be substantially insensitive to 200mM Na acetate (200 mM Na acetate vs Ctrl M9, pH7, p>0.999), confirming that the aversive effect elicited by acetic acid is due to H^+ exposure. The mean was calculated from data collected from repeats of the same experiment conducted independently across different days. In each experiment, a single worm was exposed to the drop only once. Data was subjected to two-way ANOVA followed by Tukey's multiple comparisons test. Each dot represents a single worm. WTN2-M9, pH3 = 410 worms ; WTN2-Ctrl = 410 worms; WTN2-Na acetate = 55 worms; *che-3(e1124)*-M9, pH3 = 174 worms; *che-3(e1124)*-Ctrl = 174 worms; *che- 3(e1124)*-Na acetate = 30 worms

Biology Open • Supplementary information

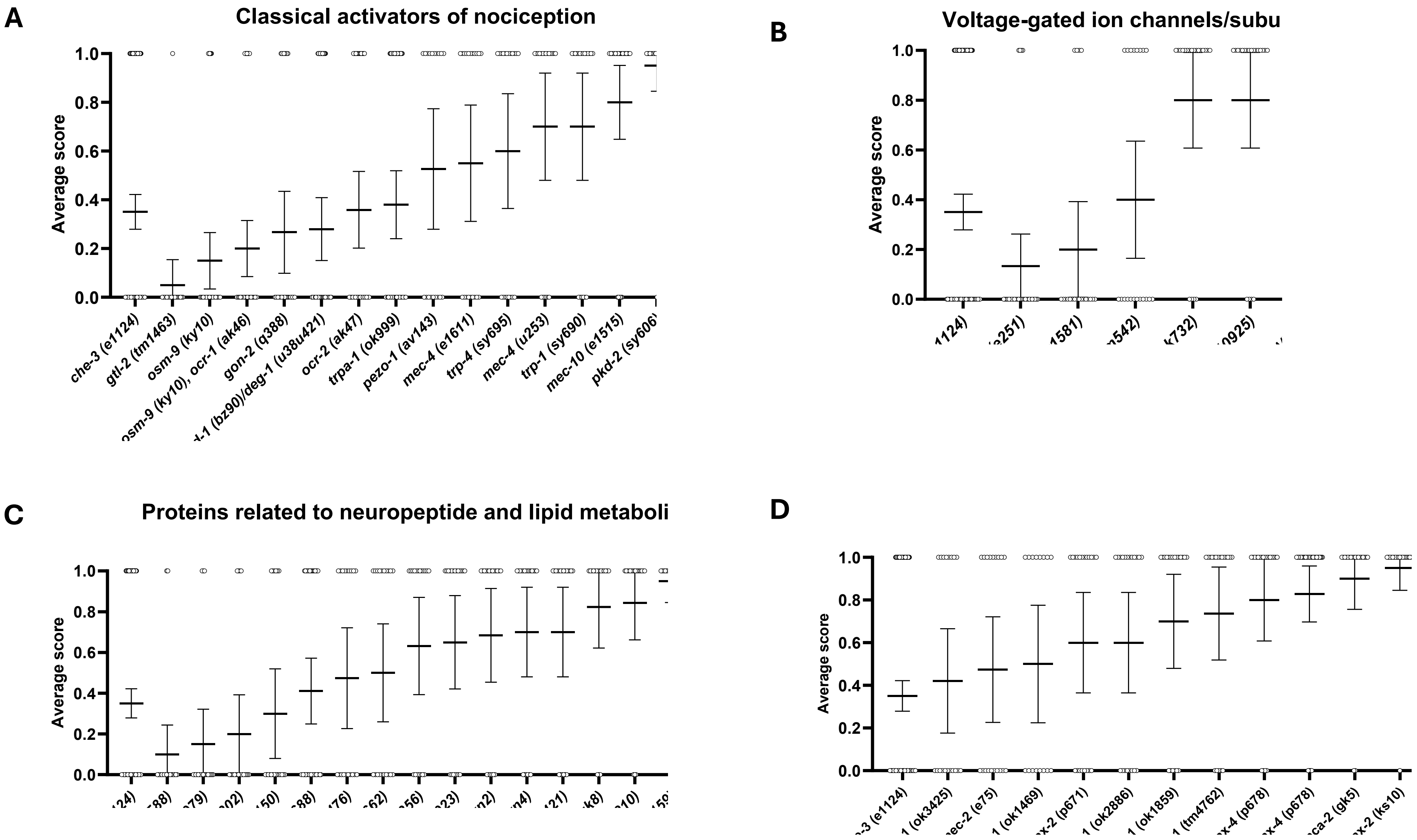

Biology Open • Supplementary information

Fig. S2. Low pH avoidance performance of *C. elegans* mutant strains for sensory detection. Scatter dot plot showing the average score of worms tested for each strain in the drop assay. Statistical analysis is shown in Figure 2 of the main text. The shape represents the distribution of responses across the strains and each dot represents an individual worm tested. *$p<0.05$, **$p<0.01$, ***$p<0.001$, **** $p<0.0001$ refers to the comparison against the WT N2 performance. Each strain was tested in at least two independent experiments.

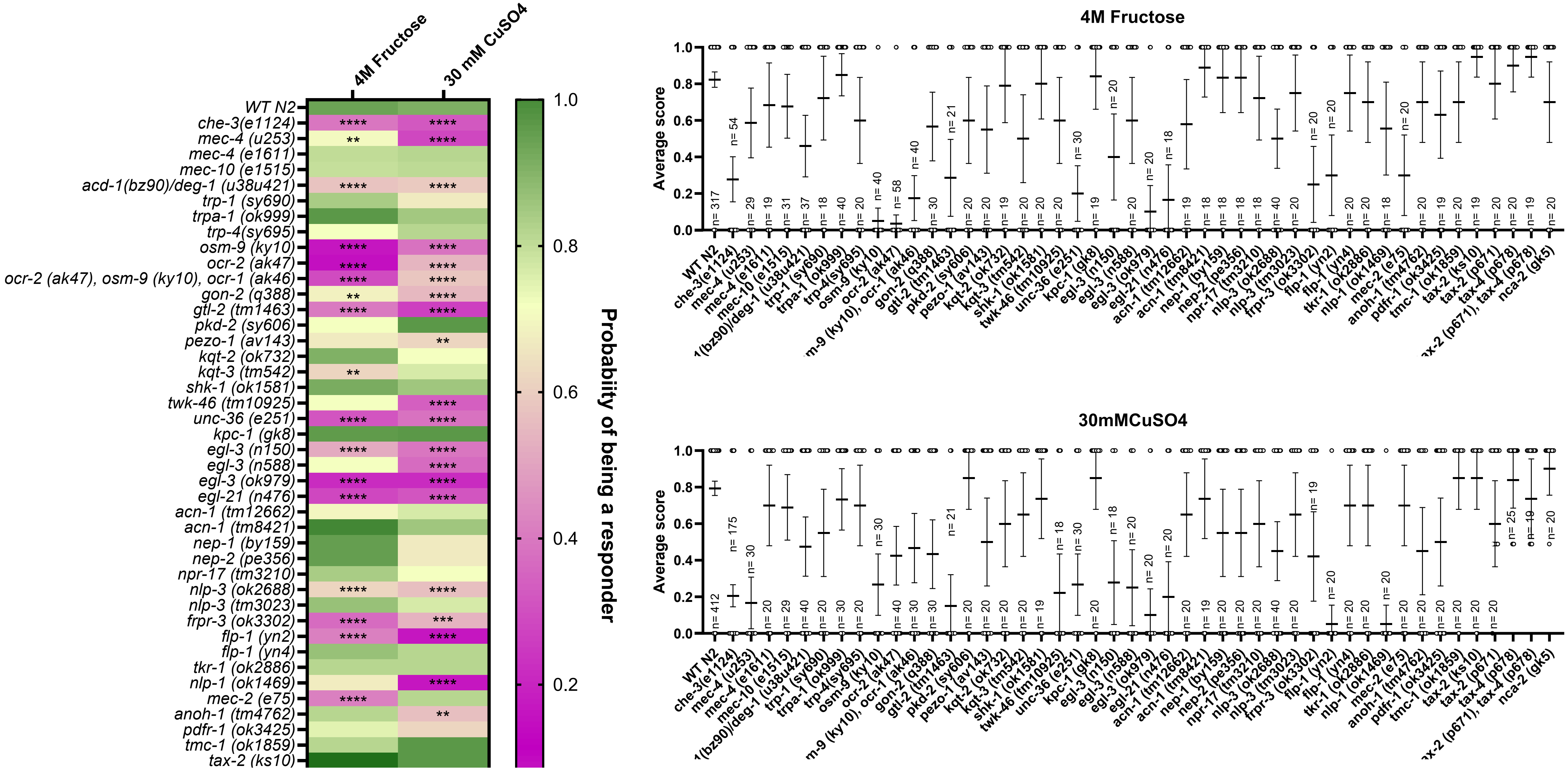

Biology Open • Supplementary information

Fig. S3. ***C. elegans*** **mutant strains were tested for aversion to other noxious cues**. On the left, heatmap summarising the average probability of being a responder (≥3 reversals within 5s) for each strain tested compared to the WT N2 performance. Significance is expressed according to the corrected p-value (q value) following Benjamini-Hochberg false discovery rate method (Q=1%). On the right, scatter dot plots representing the average score ± SD for the same data. Each dot represents an individual worm (the total is also indicated by n). Each strain was tested in at least two independent experiments.

Biology Open • Supplementary information

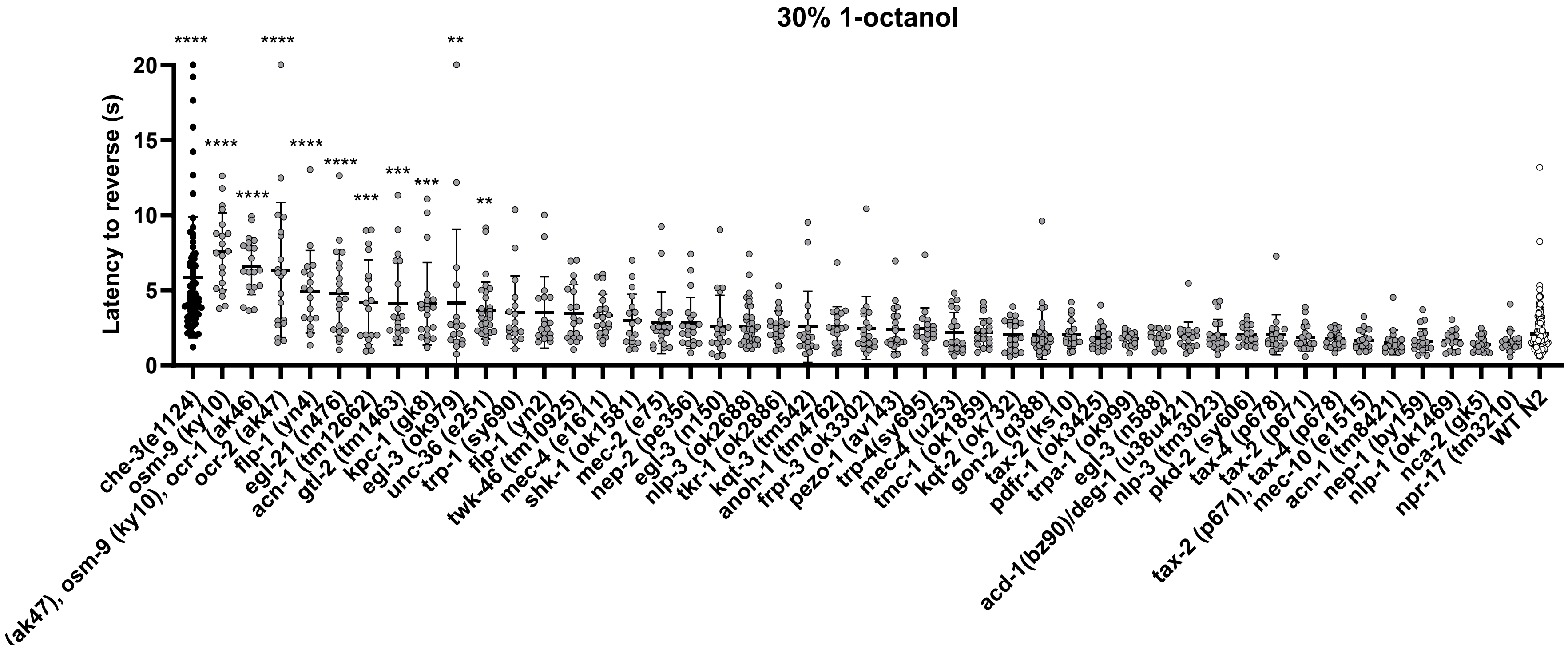

Fig. S4. *C. elegans* mutant strains were tested for volatile aversion. Scatter dot plot showing the worm average latency to reverse (s) ± SD following exposure to the volatile compound 30% 1-octanol. Data were analysed using a one-way parametric ANOVA analysis and comparing the response of each mutant to the WT N2 performance. ** p<0.01, *** p<0.001, **** p<0.0001 Each dot represents the individual animal tested. Each strain was tested in at least two independent experiments.

Biology Open • Supplementary information

Table S1.

Available for download at
https://journals.biologists.com/bio/article-lookup/doi/10.1242/bio.062268#supplementary-data

Table S2.

Available for download at
https://journals.biologists.com/bio/article-lookup/doi/10.1242/bio.062268#supplementary-data

Table S3.

Available for download at
https://journals.biologists.com/bio/article-lookup/doi/10.1242/bio.062268#supplementary-data
